# RNA Interference for Functional Genomics and Improvement of Cotton (*Gossypium* sp.)

**DOI:** 10.3389/fpls.2016.00202

**Published:** 2016-02-22

**Authors:** Ibrokhim Y. Abdurakhmonov, Mirzakamol S. Ayubov, Khurshida A. Ubaydullaeva, Zabardast T. Buriev, Shukhrat E. Shermatov, Haydarali S. Ruziboev, Umid M. Shapulatov, Sukumar Saha, Mauricio Ulloa, John Z. Yu, Richard G. Percy, Eric J. Devor, Govind C. Sharma, Venkateswara R. Sripathi, Siva P. Kumpatla, Alexander van der Krol, Hake D. Kater, Khakimdjan Khamidov, Shavkat I. Salikhov, Johnie N. Jenkins, Abdusattor Abdukarimov, Alan E. Pepper

**Affiliations:** ^1^Center of Genomics and Bioinformatics, Structural and Functional Genomics, Academy of Sciences the Republic of Uzbekistan, Ministry of Agriculture and Water Resources the Republic of Uzbekistan and “Uzpakhtasanoat” AssociationKibray, Uzbekistan; ^2^Laboratory of Plant Physiology, Wageningen UniversityWageningen, Netherlands; ^3^Crop Science Research Laboratory, United States Department of Agriculture – Agricultural Research Service, StarkvilleMS, USA; ^4^Plant Stress and Germplasm Development Research, United States Department of Agriculture – Agricultural Research Service, LubbockTX, USA; ^5^Crop Germplasm Research Unit, United States Department of Agriculture – Agricultural Research Service, College StationTX, USA; ^6^Department of Obstetrics and Gynecology, University of Iowa Carver College of Medicine, Iowa CityIA, USA; ^7^Department of Biological and Environmental Sciences, Alabama A&M University, NormalAL, USA; ^8^Dow AgroSciences LLC, IndianapolisIN, USA; ^9^Agricultural and Environmental Research, CaryNC, USA; ^10^Department of Biology, Texas A&M University, Colleges StationTX, USA

**Keywords:** antisense, cotton pest control, disease resistance, fiber quality, *Gossypium*, gene silencing

## Abstract

RNA interference (RNAi), is a powerful new technology in the discovery of genetic sequence functions, and has become a valuable tool for functional genomics of cotton (*Gossypium* sp.). The rapid adoption of RNAi has replaced previous antisense technology. RNAi has aided in the discovery of function and biological roles of many key cotton genes involved in fiber development, fertility and somatic embryogenesis, resistance to important biotic and abiotic stresses, and oil and seed quality improvements as well as the key agronomic traits including yield and maturity. Here, we have comparatively reviewed seminal research efforts in previously used antisense approaches and currently applied breakthrough RNAi studies in cotton, analyzing developed RNAi methodologies, achievements, limitations, and future needs in functional characterizations of cotton genes. We also highlighted needed efforts in the development of RNAi-based cotton cultivars, and their safety and risk assessment, small and large-scale field trials, and commercialization.

## Introduction

Today, the world demands and uses more cotton (*Gossypium* sp.) fiber than ever before. Global business revenue stimulated by cotton production is estimated at US$500 billion per year (Chen et al., 2007; Abdurakhmonov et al., 2012a) making cotton the most economically important value-added crop. Cotton is grown worldwide in more than 80 countries (Sunilkumar et al., 2006; Abdurakhmonov et al., 2012a) on 32–34 million hectares with annual total production of 25.65 million metric tons (MMTs; United States Department of Agriculture [USDA] Report, 2013). Uses of cotton fiber vary widely (Chen et al., 2007; Zhang et al., 2008; Campbell et al., 2010) from a large clothing and household usage, to numerous industrial items that account for many thousands of bales. In addition to the cotton fiber, cottonseed products (meal and hulls) are used for livestock feed, and industrial lubricants (Campbell et al., 2010) as well as a fertilizer. Cottonseed oil is used as an ingredient in food products as well as a premium cooking oil and salad dressing. Cotton stalks and leaves are used as organic matter to enrich soil and cotton stalks have been widely used as a firewood for primary energy in cooking process in rural areas as well as conversion to biogas or composting to bio-fertilizers (Federation of Indian Chambers of Commerce and Industry [FICCI] Report, 2012).

Genome of allopolyploid cotton (*Gossypium* sp.) is very complex and poorly studied. To date, cotton lags behind many other crops in studies and gains related to genomics and genetics as well as applications of marker-assisted selection (MAS) due to existence of low level of molecular polymorphisms (Abdurakhmonov et al., 2008b, 2009, 2012a) among cultivar germplasm caused by a “genetic bottleneck” during cotton domestication (Iqbal et al., 2001). The complexity of cultivated allotetraploid cotton genomes is due to the high levels of gene redundancy because of several genome duplications events before and after allopolyploidization (Adams et al., 2003). Allopolyploidization had induced a natural gene silencing, organ specific, and homoeologously biased expression of genes regulated at developmental and epigenetic level (Adams et al., 2003, 2004; Adams and Wendel, 2005; Keyte et al., 2006; Chaudhary et al., 2009). Recent studies reported that a whole genome duplication (WGD) of ancestor-like diploid cotton *Gossypium raimondii* (D5) had occurred approximately 60 million years ago (MYA) with five- to six-fold ploidy increase (Renny-Byfield et al., 2014). The gene expression analyses of this first sequenced cotton genome demonstrated complex, tissue-specific differential gene expression patterns and the diversification of gene expression level before divergence (Renny-Byfield et al., 2014) suggesting that even more complexities may be evident among cultivated allotetraploid genomes.

The allotetraploidization of A and D genome diploids occurred about 1.5-2 MYA that resulted in five distinct genomes (Adams et al., 2003; Chen et al., 2007; Zhang et al., 2008), out of which there are two allotetraploid species of cultivated cotton *Gossypium hirsutum* (so called Upland cotton) and *Gossypium barbadense* [so called Extra Long Staple (ELS) or Pima/Sea Island cotton; Abdurakhmonov et al., 2012a]. Being grown in more than 90% of world cotton growing area (Campbell et al., 2010), Upland cotton cultivars are demanded for their combination of exceptional yield, early maturity and other agronomic traits as well as moderately good fiber properties. In contrast, Pima or ELS cotton is grown in less than 5% of worldwide cotton growing area, and it is well-known for its exceptionally good fiber qualities (Liu et al., 2015a) with relatively lower yield and less desirable agronomic traits such as longer growing time, susceptibility to various diseases, and requirement of more water. One primary objective of worldwide cotton breeding effort has been to transfer ELS fiber quality into Upland cotton while keeping their early maturity, resistance, and productivity of Upland genotypes (Abdurakhmonov et al., 2012b, 2014). However, traditional breeding successes have been minimal over past century of worldwide breeding, which suffered from linkage drag and distorted segregation that occurred in interspecific hybrid progenies from Upland and Pima sexual crosses (Saha et al., 2012). Besides, simultaneous improvement of fiber quality, early flowering, early maturity, and productivity in Upland cotton (*G. hirsutum*) is a very difficult task using conventional breeding methods due to existence of negative correlations between major fiber quality and yield or maturity traits (Miller and Rawlings, 1967; Zhang et al., 2011; Abdurakhmonov et al., 2014). This challenge perhaps is even more severe under above-mentioned complexities of cotton genome, including higher rates of transgressive, differential and novel gene expression patterns, and homoeologous gene silencing (Yoo et al., 2013) that make it difficult to achieve a breeding goal.

Despite this complexity, there is a need to develop Upland cotton cultivars with increased yield, early maturity, and disease and pest resistance while producing longer and stronger fibers to be competitive with synthetic fibers in the global market. Cotton must overcome challenges from manmade fiber and become sustainable and environmentally “friendly” with reduced costs and hazards associated with chemical control methods. Novel genes must be discovered and deployed to combat salt, drought, and heat stresses as well as ever-evolving pathogens and pests such as *Verticillium/Fusarium* fungi, nematodes, and viruses (Hake, 2012; Abdurakhmonov, 2013). This prompted the cotton research community to develop an ‘innovative new generation crop technology’ to address this largely eluded and fundamentally longstanding challenge in worldwide cotton improvement programs. One of such innovative approaches is the expanded utilization of gene silencing techniques such as RNA interference (RNAi). RNAi helps to identify functions of genes with agricultural importance, and thus, address the cotton farming challenges via creating “biotechnology-derived” cotton cultivars (“biotech cotton”) with the suppression of undesirable genes but improved expression of desired trait(s) of interest. A survey of literature revealed that to date, RNAi has been applied for the functional studies of many agronomically, biologically, and physiologically important cotton (*Gossypium* sp.) genes related to the cotton fiber development; early maturity and flowering; increased yield potential; fertility and embryogenesis; viral, fungal, and insect pest resistance; tolerance to various abiotic stresses; and cottonseed and oil quality improvements.

**FIGURE 1:**
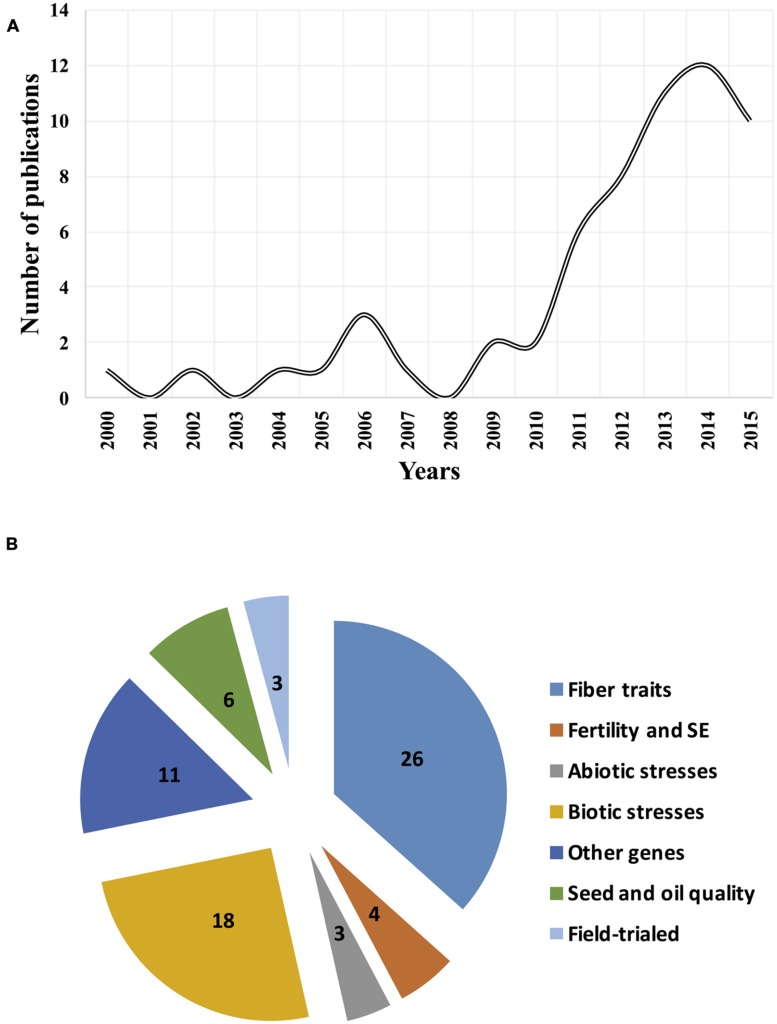
**Dynamics of publications and thematic coverage of RNA interference (RNAi) studies in *Gossypium* sp.** **(A)** Number of publications for the past 15-years period; and **(B)** RNAi studies on the targeted cotton genes or traits.

Application of RNAi is rapidly expanding due to the advances made in cotton genomics. Efforts should be expanded further with the completed genome sequencing efforts of two ancestor-like diploid D_5_ (Paterson et al., 2012; Wang et al., 2012) and A_2_ (Li et al., 2014) cottons as well as two cultivated allotetraploid Upland [*G. hirsutum* L. (AD)_1_ cv. Texas Marker-1 (TM-1); Li et al., 2015a; Zhang et al., 2015] and ELS [*G*. *barbadense* (AD)_2_ cv. Xinhai21; Liu et al., 2015a] cotton genotypes. Searching PubMed (http://www.ncbi.nlm.nih.gov/pubmed) database with the unquoted keywords of “*Gossypium gene silencing*” and “*Gossypium RNA interference*” found almost 70 refereed journal publications that experimentally cover the above-mentioned RNAi-related topics (**Figure 1**). Here, we highlighted these efforts in detail, discussing successes, existing methods, cultivar development, field trials, safety, risks, limitations, and future perspectives of RNAi in cotton research and breeding.

## RNAi Methods in Cotton

RNA interference, known as “co-suppression,” “quelling,” and/or “post-transcriptional gene silencing (PTGS),” is an evolutionarily conserved, double-stranded RNA-dependent, universal eukaryotic process to silence the expression of genes in a sequence-specific manner (Napoli et al., 1990; Romano and Macino, 1992; Hannon, 2002; Roberts et al., 2015). RNAi is induced by exo- or endogenous (i.e., micro RNAs) double stranded RNA (dsRNA) molecules. RNAi is part of the normal cellular function as well as an immune response against foreign nucleic acid signatures from viral infections (Roberts et al., 2015). In the cells, the dsRNAs are recognized by the Dicer family of enzymes and cleaved into short double stranded fragments of ∼21–25 bp long siRNAs (Hannon, 2002; Roberts et al., 2015). Further, siRNAs are separated into two single-stranded ‘passenger’ and ‘guide’ RNAs. The guide strand is incorporated into the RNA-induced silencing complex (RISC), which triggers the recognition and digestion of complementary RNA sequence signatures, whereas the passenger strand gets degraded. RISC has enzymatic digestion activity, which consists of the key components of argonaute (AGO) and P-element induced wimpy testis (PIWI) proteins (Hannon, 2002; Jakymiw et al., 2005). AGO/PIWI proteins are considered to be the critical sites for RNAi and these proteins localize within the specific P-body regions in the cytoplasm (Jakymiw et al., 2005; Sen and Blau, 2005). RISC and downstream RNAi machinery are common for exogenous and endogenous dsRNA recognition and target sequences digestion; however, there are clear differences in distinctly processing and handling of exogenous and endogenous dsRNAs (Hannon, 2002).

The regulation of gene activity of cells at the PTGS level is the main biological function of RNAi, which occurs either by the inhibition of translation of mRNA or by direct degradation of the mRNA (Hannon, 2002). Moreover, RNAi pathway components (i.e., Dicer, siRNA, and RISC) may contribute to maintenance of genome organization and structure through RNA-induced histone modification, which may silence gene activity at the pre-transcriptional level (Holmquist and Ashley, 2006). At the same time, small dsRNAs may also possibly up-regulate expression of genes through binding into a promoter region and histone demethylation (Li et al., 2006).

Usually, RNAi can be induced by the expression of antisense RNA, dsRNA and by virus induced gene silencing (VIGS) constructs. A stable RNAi in cotton is achieved by employing hairpin (HP) RNAi binary *Agrobacterium* vector constructs, which produce dsRNAs that induce RNAi machinery. The majority of cotton RNAi studies highlighted here utilized HP-mediated gene silencing. For constructing HP vectors, usually 200–500 bp long fragments of gene of interest are used. One limitation of these HP constructs is that they sometimes could generate “off-target” gene silencing due to the generation of multiple variants of short interfering RNA (siRNA) from inserted target gene fragments. This is particularly challenging when there are several paralogous, orthologous, and highly similar gene family members in an allotetraploid genome like cultivated cotton. To address this issue, researchers have developed short synthetic oligonucleotide cassette binary vectors, consisting of 19–24 bp highly specific target-gene or sRNA/miRNA sequence with intronic loop size of 7–9 bp. These synthetic oligonucleotide-based vectors could efficiently and selectively silence target genes in plants (Higuchi et al., 2009) including cotton (Abdukarimov et al., 2011). However, both HP and synthetic oligonucleotide vectors require genetic transformation where many genotypes of cotton are recalcitrant to genetic transformation. Therefore, there is a need for rapid *in vitro* and *in vivo* testing of RNAi effects for targeted genes.

Tang et al. (2004) experimentally tested the silencing power of siRNA designed for targeting the green fluorescent protein (*GFP*) gene. The silencing effects of two *GFP* derived siRNAs were tested *in vitro* cultured *GFP* transgenic cells of rice, cotton, Fraser fir, and Virginia pine. These efforts resulted in efficient silencing of *GFP* marker gene expression, which is useful for large-scale screening of gene function and drug target validation (Tang et al., 2004). Due to the limitation of siRNA delivery *in vitro* experiments, that affect the efficiency of RNAi, researchers later developed the first efficient delivery system of siRNA to plant cells including cotton by a nanosecond pulsed laser-induced stress wave (LISW). This LISW-mediated siRNA delivery system was found to be a reliable and effective method for inducing PTGS efforts *in vitro* cultured cells (Tang et al., 2006).

Further, to perform a high throughput, rapid functional validation of cotton genes and phenotypic effects researchers developed potent RNAi assays using VIGS, facilitating transient PTGS. For example, because of the important role in viral disease symptom modulation in cotton leaf curl virus (CLCV) disease, VIGS-mediated RNAi was utilized to affect betasatellite DNA of CLCV of Multan (CLCuMV). When inoculated, such VIGS system showed efficient silencing of the target genes in tobacco, *Arabidopsis* and Upland cotton plants (Kumar et al., 2014).

A variety of *Agrobacterium*-mediated VIGS vectors bearing various marker genes to monitor RNAi efficiency were developed rapidly to test the gene function of interest from cotton genome. Examples include tobacco rattle virus (TRV) vector with *GrCLA1*, *GaPDS*, and *GaANR* or fused *GaPDS*/*GaANR* marker genes (Gao et al., 2011b; Gao and Shan, 2013; Pang et al., 2013) resulting in albino or brownish plant phenotypes. In addition to the TRV-VIGS vector, a cotton leaf crumple virus (CLCrV)-based vector was developed recently and was shown as an effective RNAi method for gene discovery in cotton (Gu et al., 2014). As described above and extensively referenced herein, VIGS has been widely used for the discovery and identification of many useful genes in cotton. For instance, RNAi of cotton phytoene synthase (*GhPSY*) using TRV-induced VIGS caused a uniform bleaching of the red color in newly emerged leaves, suggesting its role in controlling red plant phenotype (Cai et al., 2014). Similarly, the VIGS induced RNAi was instrumental to discover the plant phenotypes of anthocyanidin reductase (*GhANR*) pathway genes of cotton involved in the biosynthesis of proanthocyanidins (PAs). In this study, the gene silencing effort has resulted in a significant increase in anthocyanins and a decrease in the PAs, (-)-epicatechin, and (-)-catechin in the stems and leaves of VIGS-infected plants. Results demonstrated the role of ANR pathway in the biosynthesis of flavan-3-ols and PAs in cotton (Zhu et al., 2015).

## Antisense Gene Silencing for Early Functional Studies of Cotton Genes

It is noteworthy to mention that the first pioneering attempts on silencing of cotton genes were performed by using antisense constructs. The pioneering effort is dated to John (1996) who studied two E6 genes isolated from Upland and Pima cottons. A 60–98% suppression of E6 activity using antisense transgenic cotton plants revealed no noticeable phenotypic changes in fiber development. This research demonstrated that E6 was not critical to the fiber development process (John, 1996). Antisense suppression of cotton small GTPase Rac (*RAC13*) genes decreased the levels of H_2_O_2_ in fiber cells, which in turn affected secondary wall formation of cotton fibers (Potikha et al., 1999). Ruan et al. (2003) successfully constructed antisense vectors for a 70% suppression of cotton sucrose synthase (*SUS*) gene that resulted in fiberless and shrunken seed phenotypes. Later, the functions of several cotton genes such as cotton steroid 5-alpha-reductase (GhDET2; Luo et al., 2007), cotton myeloblastosis (*GhMYB109*; Pu et al., 2008), and *GhPEL* gene, encoding a pectate lyase (Wang et al., 2010a) were studied using antisense technology, which resulted in significant reduction of fiber elongation and shorter fibers. These results have suggested important roles of these genes in fiber development.

Antisense technology was also utilized to regulate other aspects of cotton besides fiber genes. Inverted-repeat-based gene constructs designed for two key cotton seed-specific fatty acid desaturase genes, *GhSAD-1* and *GhFAD2-1*, resulted in increased levels of stearic and oleic acids in RNAi cotton lines, respectively. Results also showed that the content of palmitic acid significantly decreased in both high-stearic and high-oleic lines, providing a promising opportunity for the development of nutritionally improved cottonseed oil (Liu et al., 2000). CLCV significantly reduces boll formation and development in cotton. Antisense vectors were constructed for important genes of CLCV with the aim of affecting the virulence of this virus. Such targeted genes were 5′ and 3′ fragments of DNA replication gene (*AC1)* as well as a transcription activator (*AC2*) and replication enhancer (*AC3*) genes. Transformation of these antisense constructs into tobacco plant helped to obtain virus resistant transgenic plants (Asad et al., 2003).

Gossypol is a general biocide that provides protection from insect predation, but it restricts the usefulness of cottonseed as a feed and protein source for human and monogastric animals. In the efforts to manipulate gossypol, Martin et al. (2003) developed and transformed cotton with an antisense construct of *CDN1-Cl*, a member of a complex gene family of delta-(+)cadinene (CDN) synthase. These efforts resulted in a reduction of *CDN* synthase gene activity and decreased up to 70% of gossypol content. Further analyses of the first generation transgenic cotton plants demonstrated a significant amount of reduction of gossypol (92%), hemigossypolone (83%) and heliocides (68%) in leaves (Martin et al., 2003) and seeds (Benedict et al., 2004), negatively influencing the biosynthesis of cadinene sesquiterpenoids and heliocides in cotton plants. Later, Townsend et al. (2005) reported that the *Agrobacterium*-mediated genetic transformation of constitutive or seed-specific antisense constructs demonstrated the suppression of *CDN1-C4* genes in a response to bacterial blight infection of cotyledons in the constitutive antisense plants, suggesting a specific role of particular cotton sesquiterpene in the bacterial blight pathogenesis in cotton (Townsend et al., 2005). Similar antisense technology was also used for improvement of cottonseed oil by Sunilkumar et al. (2005) where authors developed the homologous alpha-globulin B promoter driven antisense construct for *FAD2* gene that reduced the expression of delta -12 desaturase in cottonseed. Efforts resulted in two-fold increase of the oleic acid level with an accompanying decrease of linoleic acids in transgenic cottonseeds.

Further, with the discovery and understanding of the mechanisms, RNAi has been applied widely in cotton research, and it has become a major research tool for studying gene functions and breeding of novel cotton cultivars. For the past 15-years, more than 60 RNAi related studies have been published in cotton where we see dramatic increase of efforts after 2010. These studies have targeted functional genomics of important cotton genes including, but not limited to the fiber development, fertility and somatic embryogenesis (SM), abiotic and biotic stress, cottonseed and oil quality genes of cotton (**Figure 1**; **Supplementary Table S1**). These efforts have resulted in the development of efficient RNAi methodologies, and RNAi-derived novel biotech cotton lines and cultivars that were subsequently utilized for the functional studies, breeding, and farming of cotton, which we have reviewed below in detail.

## RNAi for Functional Genomics of Fiber Genes and Quality Improvement

Majority (37%) of RNAi published studies have targeted the study of functional aspects of fiber-related genes in cotton (**Figure 1B**). Cell elongation and cell cytoskeleton factors are important for fiber development. One of the first fiber-related RNAi efforts targeted the actin cytoskeleton (*GhACT*) genes that express during fiber development. RNAi of *GhACT1* significantly repressed the expression of the target and disrupted the actin cytoskeleton network in fibers, resulting in the inhibition of fiber elongation. Researchers concluded that *GhACT1* plays an important role in fiber elongation but not fiber initiation (Li et al., 2005). Because cotton fibers are seed epidermal cells and resemble epidermal trichomes of leaves, researchers targeted the characterization of orthologs of plant leaf trichome initiation factors in cotton fibers. Two highly similar cotton *GhMYB25* genes were characterized in allotetraploid cotton, which were acquired from A and D genome ancestors. RNAi silencing of both *GhMYB25* genes using single hairpin (HP) construct altered the timing of rapid fiber elongation and resulted in short fibers, dramatic reduction in trichomes on other parts of the plant, and a reduction in seed production. These results demonstrated the important functional role of *MYB* genes in cotton fiber and seed development (Machado et al., 2009).

Later, Walford et al. (2011) characterized *GhMYB25*-*like* cotton *MYB* gene with *R2R3* domain from a fiberless mutant of cotton (Xu142 *fl*). RNAi of *GhMYB25*-*like* gene caused fiberless cottonseeds in transformed cotton plants. However, normal trichome development occurred in other parts of the cotton plant, similar to the Xu142 *fl* mutant lines. Like Xu142 *fl* mutants, *GhMYB25*-*like* RNAi plants had a lowered expression of fiber-specific *MYBs* including *GhMYB25* and *GhMYB109*; however, RNAi of both *GhMYB25* and *GhMYB109* did not change the expression level of *GhMYB25*-*like*. This suggested that *GhMYB25*-*like* is upstream from those *MYBs* and important factor for cotton fiber development (Walford et al., 2011). Another trichome and early fiber expressed *MYB*-like gene with a putative homeodomain leucine zipper (HD-ZIP) transcription factor (*GhHD-1*), functioning downstream of *GhMYB25*-*like* gene, was characterized in cotton. RNAi suppression of *GhHD-1* decreased trichome formation and delayed the timing of fiber initiation. *GhHD-1*-derived RNAi plant analyses proved that this gene conditions the levels of ethylene and reactive oxidation species (ROS) through a WRKY transcription factor and calcium-signaling pathway genes (Walford et al., 2012). Similarly, RNAi of another cotton HD-ZIP transcription factor (*GhHOX3*) gene greatly (>80%) reduced fiber length, whereas its overexpression led to longer fibers. Results of this recent report elucidated a novel mechanism of transduction of gibberellic acid signal by a homeodomain protein to promote fiber cell elongation (Shan et al., 2014).

Arabinogalactan proteins (AGPs) are involved in many aspects of plant development. Researchers reported the essential roles of fasciclin-like arabinoglactan (*FLAs*) genes in cotton fiber development using *GhAGP4* RNAi-derived transgenic plants. Results revealed significant suppression of target genes as well as partial suppression of the expression of other *FLA* related genes, resulting in an inhibition of fiber initiation and fiber elongation processes. The resulting RNAi plants had significantly shorter fibers and inferior fiber quality parameters due to disruption of the cytoskeleton network and the cellulose deposition in fiber cells (Li et al., 2010). Subsequently, *GhFLA1* was identified in cotton, and it was demonstrated that its suppression slowed down fiber initiation and elongation. As a consequence, plants produced significantly shorter fibers compared to the wild type controls (Huang et al., 2013). These results demonstrated the importance of cotton *FLA* genes in fiber length and quality improvements.

Another cell elongation and expansion factor of cotton, characterized using RNAi approach, was vacuolar invertase (*GhVIN1*). *GhVIN1* activity was significantly higher in fiber compared to leaf, stem, and root. It expressed at subtle level in fiberless cottonseed epidermis. Interestingly this gene started expressing in initiating fibers and reached a high-level in elongation phase, but dramatically dropped after the elongation. RNAi plants for *GhVIN1* gene efficiently suppressed the transcript and negatively affected the elongation, while overexpression of *GhVIN1* showed positive changes in fiber elongation rates. It was demonstrated that *GhVIN1* regulates fiber elongation in an osmotic dependent manner (Wang et al., 2010b). Further, RNAi- induced down-regulation of *GhVIN1* expression has generated a dosage-dependent fiberless cottonseed phenotype without influencing seed development, which was due to the markedly reduction of VIN activity (Wang et al., 2014). The results of Wang et al. (2014) have suggested that *GhVIN1* mediates hexose signaling, which is important for the regulation of key regulatory genes of fiber cell initiation and differentiation from the ovule tissue in early stage of fiber development.

RNA interference approach was also instrumental in analyzing the function of a cotton *PROTODERMAL FACTOR1* gene (*GbPDF1*), where its RNAi caused a retardation of fiber initiation, produced shorter fibers and lowered the lint percentage compared to the wild-type controls. These results demonstrated that *GbPDF1* is an important genetic factor, positively conditioning cotton fiber development. The detailed characterization of *GbPDF1* RNAi plants revealed a higher accumulation of hydrogen peroxide, and reduced expression of ethylene and pectin synthesis or sugar transport related genes during fiber development. These RNAi study results suggested that “*GbPDF1 plays a critical role together with interaction partners in hydrogen peroxide homeostasis and steady biosynthesis of ethylene and pectin during fiber development via the core cis-element HDZIP2ATATHB2*” (Deng et al., 2012).

Because of their single celled-expansion-model property, developing cotton fibers have been used to elucidate cell wall formation using various transgenomic tools, including VIGS and high throughput modern experimental technologies (Haigler et al., 2012). Researchers extended TRV triggered VIGS in cotton leaves to study the function and biological role of a microtubule-severing protein (KATANIN) and a positive regulator of lipid biosynthesis (*WRINKLED1*) genes in cotton fiber development. RNAi plants having a decreased expression of *KATANIN* gene had shorter fibers and increased cottonseed oil in endosperm, whereas RNAi suppression of *WRINKLED1* resulted in increased fiber length with reduced cottonseed oil level. These results not only demonstrated the possibility to increase fiber length by repartitioning carbon flow but also highlighted the effectiveness of the TRV-VIGS method for rapid functional analysis of genes involved in cotton fiber development (Qu et al., 2012). A recent study has demonstrated that the use of VIGS constructs with several cell wall membrane lipid genes of cotton encoding fatty acid desaturases [*GhΔ(15)FAD*], phosphatidylinositol synthase (*GhPIS*), or phosphatidylinositol kinase (*GhPIK*) resulted in significant decrease of fiber length (Liu et al., 2015b).

RNA interference has also been utilized to annotate the function of several transcription factor genes involved in fiber development. For instance, RNAi suppression of a class I TCP transcription factor (*GbTCP*) yielded shorter fiber, a reduced lint percentage, and a lower fiber quality than the wild-type plants. Results from this study suggested that the modulation of jasmonic acid (JA) biosynthesis/response as well as other pathways such as ROS, calcium channel, and ethylene signaling by *GbTCP* gene is important for fiber and root hair development (Hao et al., 2012). Early attempts of studying the roles of sucrose synthase (*SUS*) genes using suppression of its activity resulted in shorter cotton fibers (Ruan et al., 2003). Recently, a novel cotton sucrose synthase (*GhSUSA1*) was characterized using germplasm analysis and genetic mapping strategies, where its RNAi-mediated suppression in transgenic cotton reduced fiber quality and decreased the boll size and seed weight sharply contrasting *GhSUSA1* overexpression events (Jiang et al., 2012). The interesting role of some metabolites in cotton fiber was recently elucidated in both overexpression and RNAi silencing experiments of flavanone 3-hydroxylase (*F3H*). *F3H* uses naringenin (NAR) metabolite as its substrate. An excess of NAR negatively affects fiber development; therefore, RNAi of *F3H* gene significantly increased the NAR content of fiber cells that caused a delay in fiber development. RNAi phenotype was transferable by hybridization to other genotypes, and appeared to be more severe in the genetic background of high-flavonoid brown fibered *G. hirsutum* T586 line. Results suggested the importance and prospective utilization of flavonoid metabolism as a novel pathway for cotton fiber improvement (Tan et al., 2013).

RNA interference experiments, targeting the *REPRESSOR OF SILENCING-1* (*ROS1*) gene with a conserved DNA demethylase domain, revealed the promotion of DNA methylation in resulting transgenic RNAi cotton lines, accompanied by a significantly reduced fiber growth (Jin et al., 2013). These results suggested that fiber development is greatly affected and under the control of epigenetic regulations. RNAi was a great help in annotating the exact function of several ‘candidate’ cotton genes in the fiber development, particularly those involved in various transport and ion exchange proteins that play a vital role. Two of these proteins are water transporter aquaporins (*GhPIP1* and *GhPIP2*) that condition fiber elongation. There is a complex hetero-oligomer *GhPIP2* protein interaction network, and RNAi cotton plants with reduced expression of *PIP2* transcript markedly delayed fiber elongation and produced shorter fibers. Results elucidated the molecular mechanism of *GhPIP2* involvement in fiber development. In conclusion, *GhPIP2* proteins are the primary aquaporin isoforms in fibers, proving their importance for rapid fiber elongation (Li et al., 2013). Similarly, RNAi of cotton Annexins [*GhAnn2*; Ca(2+)- and phospholipid-binding proteins] significantly retarded fiber elongation and secondary cell wall synthesis, resulting in shorter and thinner mature fibers in RNAi plants. This primarily was due to decreased level of the rate of Ca(2+) influx at the fiber cell apex of *GhAnn2* RNAi lines suggesting possible fiber development modulation through Ca(2+) fluxes and signaling (Tang et al., 2014). Further, RNAi of cotton α-expansin genes *GbEXPA2* and its truncated version *GbEXPATR* also resulted in shorter fibers with thicker cell walls sharply contrasting the results of their overexpression. These results fueled a hope for development of cultivars with improved fiber (Li et al., 2015b).

In contrast, RNAi of a proline-rich protein of cotton (*GhPRP5*) enhanced fiber development resulting in longer fibers in RNAi cotton plants compared to wild type controls (Xu et al., 2013) opening a promising venue to develop agriculturally valuable superior fiber RNAi cotton cultivars. Abdurakhmonov et al. (2014) reported a similar effect when cotton phytochrome A1 (*PHYA1*) gene was repressed by using RNAi, which resulted in improvement of fiber quality. Because of known influence of red/far-red light ratio on the fiber length researchers investigated RNAi of the cotton *PHYA1* gene that drastically reduced *PHYA1* gene transcript up to ∼70%, but caused a compensatory overexpression of remaining phytochrome genes by up to ∼20-fold in somatically regenerated *PHYA1* RNAi cotton plants. RNAi of *PHYA1* gene significantly enhanced the upper half-mean fiber length and improved other major fiber characteristics without adverse effects to the other agronomically important traits such as maturity and yield. RNAi phenotype expression related to fiber quality, root and vegetative growth, and early flowering was genetically stable, heritable, and transferable. Importantly, RNAi of *PHYA1* gene increased seed cotton by 10–17% when compared with the controls in replicated field plot experimental trials (Abdurakhmonov et al., 2012b, 2014). As a result of this effort, the first generation RNAi cultivars were developed using state-of-the-art RNAi technology, which are the world’s first biotech cotton cultivars with improved fiber quality and other key agronomic traits without adversely affecting the yield (Abdurakhmonov et al., 2014; Cotton Outlook, 2014; Macron, 2014).

## RNAi for Biotic Stress Studies

As for fiber development studies, RNAi has been a golden tool to study biotic resistance aspects in cotton. Twenty five percent of all published RNAi studies (**Figure 1B**) have resulted in many successful applications for functional analyses of viral, fungal, and insect resistance genes as well as for developing improved cotton cultivars.

### RNAi for Functional Genomics of Fungal Resistance

*Verticillium dahliae* and *Fusarium oxysporum* are two species of wilt disease-causing fungal pathogens of cotton that globally pose a huge threat for cotton farming and production. RNAi has been successfully applied to study wilt disease resistance aspects and develop wilt-resistant cotton lines. Using VIGS-mediated RNAi approach, researchers demonstrated silencing of *GhNDR1* and *GhMKK2* genes that decreased *Verticillium* wilt resistance in cotton (Gao et al., 2011a). Similarly, VIGS of *Verticillium* wilt-induced *GbVE1* gene isolated from wilt resistant Pima (*G. barbadense*) decreased resistance to this fungal pathogen in contrast to its overexpression (Zhang et al., 2012). Results suggested that the *GbVE1* gene is one of the candidate genes to develop wilt resistant cotton cultivars through modern biotechnology and conventional breeding. VIGS applied for subfamily of SM receptor kinases genes *GhSERK*/*GhBAK1* (Gao et al., 2013a), a key gossypol biosynthesis enzyme gene *GbCAD1*, and an important regulatory *GbSSI2* gene (functioning in the crosstalk between salicylic acid and JA signal pathways; Gao et al., 2013b) revealed functional importance of these genes in *Verticillium* wilt resistance of cotton with decreased resistance when they were silenced (Gao et al., 2013a).

Zhu et al. (2013) characterized the ghr-miR482 family in cotton and demonstrated its down-regulation in cotton seedlings of a susceptible line after infection with *Verticillium* wilt, which caused up-regulation of several nucleotide binding site-leucine-rich-repeat (NBS-LRR) target genes. This provided evidence that like other plants, cotton has similar miRNA-mediated gene regulation mechanism that release control over the expression of NBS-LRR defense genes upon fungal pathogen attack. Heterologous VIGS, using a conserved fragment from the enhanced disease susceptibility 1 (*GbEDS1*) gene of cotton in *Nicotiana benthamiana* plants, increased susceptibility to *Verticillium* wilt. Therefore, *GbEDS1* could be a novel biotechnological tool in the regulation of cotton defense against *V. dahliae* (Su et al., 2014). Mitogen-activated protein kinase (*MAPK*) cascade genes are considered important in plant growth, development, and stress responses. The *MAPK* gene family of cotton, consisting of 28 putative members, has been characterized and phylogenetically clustered into the four known A, B, C and D groups, with more *MAPKs* containing the TEY phosphorylation site (18 members) than the TDY motif (10 members). Furthermore, TRV-VIGS approach has helped to discover important roles of *MAPKs* in diverse functions in cotton, including various developmental stages of vegetative and reproductive growth and in the biotic stress response. It was shown that VIGS of *MPK9*, *MPK13*, and *MPK25* could significantly enhance cotton susceptibility to the *Verticillium* wilt pathogen (Zhang et al., 2014).

Availability of detailed plant genome information and its combination with a rapid gene function detection using VIGS and heterologous expression facilitated a global level of gene function screening in plants including cotton. Recently, using this approach, Xu et al. (2014) screened 38 cotton genes for their response to *V. dahliae* and jasmonate treatment. Twenty-four of these genes were found to be differentially regulated by pathogen inoculation and most of these have positively responded to both *Verticillium* infection and JA stimuli. The results of functional analysis suggest that reactive oxygen species, salicylic acid- and JA-signaling pathways are involved in disease resistance response of cotton against *V. dahliae*, highlighting the power of VIGS in cotton functional genomics and data mining (Xu et al., 2014).

Very limited information is available on RNAi directed studies in understanding the resistance of *Fusarium oxysporum* (FOV) wilting pathogen that has been a serious threat to cotton production across the world (Abdullaev et al., 2015). With the target to develop novel RNAi-based cotton cultivars against FOV, Shapulatov et al. (2015) recently characterized a “top-layer” of small RNA/miRNA profile in FOV race 3 infected versus uninfected cotton seedlings of resistant and susceptible cotton genotypes. This study identified many small RNA and miRNA signatures that expressed differentially and targeted key proteins associated with disease resistance (Shapulatov et al., 2015) that could be useful for future RNAi studies.

### RNAi for Insect Resistance and Pest Control

Conventionally, cotton pests are controlled by using chemically derived insecticides, which have been later expanded with the *Bacillus thuringiensis* (Bt) *Cry* toxin gene expressed in transgenic crops (Qiu et al., 2015). Bt-crops significantly helped to combat major agricultural insects and decreased the pesticide utilization in many crops including cotton. There is no doubt that Bt-cotton brought significant economic and environmental benefits; however, currently the examples exist where insect resistance has arisen against the Bt toxins in several crops, including cotton. This prompted the plant research community to explore alternative approaches for insect pest control.

In this context, RNAi appeared as an alternative approach to control insect pests based on findings in *Caenorhabditis elegans* where feeding nematodes with dsRNAs, produced by *Escherichia coli*, could cause silencing of expression of targeted gene (Roberts et al., 2015). Although the mechanism of dsRNA uptake, respond, and efficiency in insects are distinct and variable compared to *C. elegans*, a constitutive expression of dsRNAs for target insect genes through the plant-mediated RNAi could trigger pest target gene RNAi when fed with plant tissues, expressing insect gene-specific dsRNAs (Roberts et al., 2015). Several important factors should be taken into consideration to apply RNAi via insect-resistant transgenic plants. These include: (1) detection of vital and specific gene of target insect whose RNAi must cause developmental retardation or lethality to the preferentially targeted insect species; and (2) dsRNAs must be uptaken by the insect midgut system from plant tissues, and they must induce silencing of the target gene, amplify rapidly and spread systemically.

The first proof of the concept of RNAi-plant mediated pest control of cotton bollworm was demonstrated by Mao et al. (2007) using dsRNA of insect-derived cytochrome P450 monooxygenase gene (dsCYP6AE14). Consequently, feeding the insects with dsCYP6AE14 expressing plant tissues increased the levels of dsCYP6AE14 transcript in the midgut of insects and decreased CYP6AE14 expression in insects. This resulted in retardation of larval growth due to intolerance to toxic gossypol as well as more sensitivity to the insecticides used (Mao et al., 2007, 2011; Tao et al., 2012). This research highlighted the power of RNAi cotton plants expressing dsCYP6AE14 that efficiently controlled cotton bollworms and developed insect-proof cotton cultivars (Mao et al., 2011; Tao et al., 2012). Further, the combined usage of dsCYP6AE14 and plant cysteine proteases, such as *GhCP1* from cotton (*G. hirsutum*) and *AtCP2* from *Arabidopsis*, have provided the increased protection from bollworm than either of the single-transgenic events. The increased effect was due to the improvement of dsCYP6AE14 uptake by insect cells because of increasing the permeability of the insect peritrophic matrix by cysteine proteases. This provided a potent plant-mediated RNAi approach against herbivorous insects (Mao et al., 2013).

A similar effort reported the potential of an alternative gene for plant RNAi-mediated insect pest control. *In vivo* RNAi of the 3-hydroxy-3-methylglutaryl coenzyme A reductase [HMG-CoA reductase (*HMGR*)] gene, a key enzyme in the mevalonate pathway in insects, effectively inhibited the fecundity and oviposition of the females, and significantly reduced vitellogenin (Vg) mRNA levels, demonstrating its potential against *Helicoverpa armigera* and other insect pests (Wang et al., 2013). In order to better understand RNAi mechanism of insects and to identify target genes for RNAi, the transcriptome database was developed for the cotton boll weevil, *Anthonomus grandis*. Several key components of RNAi machinery (PAZ domain and SID-like proteins) and key insect genes (e.g., chitin synthase 1) for inducing RNAi have been characterized. Further, *A. grandis* female adults exposed to dsRNA of a chitin synthase gene produced non-viable eggs and malformed alive larvae (Firmino et al., 2013). Further, RNAi has been shown to be a very valuable tool to identify key molecular receptors providing efficiency of widely used Bt-crops through improvement of susceptibility to Cry toxins. An oral RNAi inducer for cadherin *SeCad1b* gene significantly decreased susceptibility to Cry1Ac and Cry2Aa in *Spodoptera exigua* larvae (Qiu et al., 2015).

## RNAi for Abiotic Stress Genes

An understanding of tolerance mechanisms to the abiotic stress (including salinity, drought, and heat), and their molecular basis have been major goals of plant research community, and are imperative to the improvement of stress tolerance, and environmental adaptation of crop plants, including cotton. RNAi methodology has been productive in characterizing the biological function of many cotton genes in abiotic stress tolerance. For instance, a VIGS-mediated sucrose non-fermenting 1- related protein kinase 2 (*GhSnRK2*) gene silencing mitigated drought tolerance in cotton plants, indicating that *GhSnRPK2* positively condition drought stress and low temperature tolerance (Bello et al., 2014).

Another VIGS-mediated RNAi study with an R2R3-type *GbMYB5* transcription factor, sharply contrasting its overexpression, decreased the tolerance of cotton plantlets to drought due to decreased proline content, antioxidant enzyme activities, and increased malondialdehyde (MDA) content in cotton under drought stress. Results from this study suggested that *GbMYB5* is an important positive factor contributing to the environmental adaptation of plants under drought stress condition (Chen et al., 2015). Further, as mentioned-above, RNAi of cotton *PHYA1* genes (Abdurakhmonov et al., 2014) generated improved drought, salt, and heat tolerance compared to wild type plant that might be due to increased photosynthesis, regulation of plant salt tolerance genes and longer, better developed root systems of *PHYA1* RNAi cotton plants (Abdurakhmonov et al., 2014).

## RNAi for Fertility and Embryogenesis Studies

Somatic embryogenesis, pollen/anther development, fertility and heterosis questions are the topics of central interest to cotton physiology, genetics and breeding as well as modern biotechnology studies. In particular, an understanding of SE in cotton is very important for genetic engineering and modern cotton breeding programs due to limitations imposed by a high degree of genotype-dependency and only a very few genotypes having regenerative capability from single cells. An RNAi approach has been particularly helpful in the study of high mobility group box (*HmgB*) family genes of cotton involved in SE. RNAi of the *GhHmgB3* gene (Hu et al., 2011) decreased level of the *GhHmgB3* in hypocotyl-derived cotton somatic cells, which rapidly dedifferentiated but did not develop into embryogenic callus (EC) tissues. When the *GhHmgB3* RNAi construct was transformed into EC cells, tissue proliferation and differentiation in transformed samples were significantly improved, but embryonic tissues did not develop into complete plantlets. Further, researchers found that RNAi of *GhHmgB3* caused a series of β-catenin-related mechanisms that might condition the deregulation of proliferation and differentiation of cells in cotton SE (Hu et al., 2011).

RNA interference approach to studying the *GhSERK1* gene, encoding a putative leucine-rich repeat receptor protein kinase (LRR-RLK) with 11 domains, generated a series of male-sterile cotton lines, including mutants with normal fertility, semi-sterility, and complete sterility. Sterility was strongly correlated with the level of RNAi. Results demonstrated that the *GhSERK1* gene has an important role in the development of anthers, especially in the formation of pollen grains (Shi et al., 2014). Recent RNAi and co-suppression mediated down regulation study toward investigating the potential function of cotton KORRIGAN (*GhKOR1*), a highly conserved membrane-bound endoglucanase, has resulted in smaller filial tissue and reduced seed weight, leading to various abnormalities in endosperm formation and delayed embryo development. Results collectively suggested that the *GhKOR1* gene is required for the development of viable cottonseed with normal seed germination and seedling growth (Shang et al., 2015). Another interesting RNAi gene silencing study targeting cotton acyl-CoA N-acyltransferase (EC 2.3; *GhACNAT*) using VIGS resulted in abnormal anther formation and sterile plants. *GhACNAT* is involved in lipid metabolism and JA biosynthesis; therefore, treatments with exogenous methyl jasmonate “*rescued anther dehiscence and pollen release in GhACNAT-silenced plants and caused self-fertility*” (Fu et al., 2015).

## RNAi for Seed and Oil Quality

Following the inverted-repeat-based gene constructs study (Liu et al., 2000), a hairpin RNA-mediated gene silencing was applied by the same research group to genetically modify the fatty acid composition of cottonseed oil (Liu et al., 2002). The target genes were two key fatty acid desaturase genes, *GhSAD-1* encoding stearoyl-acyl-carrier protein delta 9-desaturase and *GhFAD2-1* encoding oleoyl-phosphatidylcholine omega 6-desaturase. RNAi of these two genes revealed similar down-regulation effects as reported earlier (Liu et al., 2000), i.e., 40 and 77% increase of stearic and oleic acids, respectively, with substantial decrease of palmitic acid content in both dsSAd-1 and dsFAD2 expressing lines. However, down-regulation was observed at lower frequencies for the antisense constructs as compared to the HP RNAi approach. Interestingly, researchers demonstrated “stacking” of both dsSAd-1 and dsFAD2 HP constructs into one cotton genotype via sexual intercrossing of independently silenced RNAi lines that resulted in the same degree of down-regulation of the target genes as observed in the individually silenced parental lines. Further, RNAi modulation of these key fatty acid genes (single or stacked) to various degrees provided an opportunity to produce cottonseed oils with novel combinations of palmitic, stearic, oleic, and linoleic acid contents. These results should help to increase cottonseed oil quality and consumer satisfaction (Liu et al., 2002).

Besides oil production, cottonseed is a rich source of proteins that “*can potentially provide the protein requirements for half a billion people per year*” (Sunilkumar et al., 2006), provided if cottonseed is free of the toxic gossypol present within the seed glands. Conventional geneticist developed gossypol-free glandless cultivars with mutations of gossypol biosynthesis pathway in entire cotton plant. Therefore, such glandless cotton cultivars were highly susceptible to insect pests limiting their wide farming. However, RNAi was of a particular help in providing an opportunity to develop seed tissue-specific, highly stable and heritable silencing of the key delta-cadinene synthase gene of gossypol biosynthesis during seed development, leaving gossypol intact in other green parts of the cotton. This breakthrough research in cotton offered a novel solution to increase food sources for sufficient nutrition for hundreds of millions of people (Sunilkumar et al., 2006). Rathore et al. (2012) further fine-tuned RNAi-knockdown of δ-cadinene synthase gene(s) and developed ultra-low gossypol cottonseed (ULGCS) cotton lines that exhibited multi-generational stable RNAi effects.

## Natural RNAi for Cotton Genes and Traits

Besides the use of RNAi for targeted inhibition of protein coding genes, it is important to understand the function of natural miRNAs present in cotton. The first ‘wet-bench’ small RNA characterization from developing cotton ovule was reported by Abdurakhmonov et al. (2008a) followed by the characterization of small RNAs in fiber development in a large scale deep sequencing efforts (Kwak et al., 2009; Pang et al., 2009; Ruan et al., 2009). Deep sequencing of small RNAs isolated from different cotton tissues has revealed the presence of evolutionarily conserved miRNAs, which target common developmental programs like organ and tissue differentiation and timing of development. Many cotton-specific miRNAs have been identified but their functions are still unclear and need experimental validation (Wang and Zhang, 2015). Natural cotton miRNAs have been studied in Upland cotton (*G. hirsutum*, AADD), Sea Island cotton (*G. barbadense*, AADD), *G*. *herbaceum* (A_1_), *G. arboretum* (A_2_), and *G. raimondii* (D_5_). Some miRNAs show differential expression under salt stress; they could therefore be potential targets to improve salt stress responses (Yin et al., 2012). Most interestingly, miRNAs may play an important role in cotton fiber development as 140 targets of 30 conserved miRNAs and 38 targets of five candidate miRNAs were identified through degradome sequencing of cotton fiber RNA (Zhang et al., 2013a; Liu et al., 2014). Targeting of one species of miRNA (miRNA156/157) resulted in the reduction of mature fiber length, illustrating the importance of natural miRNAs in cotton.

Recently, several cotton micro RNA (miRNA) sequence signatures have been identified (Shweta and Khan, 2014) that efficiently target and stop the replication of Allahabad CLCV (CLCuAV) genome. The ghr-miR2950 was capable of targeting all the viral genes while ghr-miR408 targeted overlapping transcripts of *AC1* and *AC2* genes. Further, researchers identified that ghr-miR394, ghr-miR395a and miR395d could bind overlapping transcripts of *AC1* and *AC4* genes, which are involved in CLCuAV viral replication; therefore, these RNAi inducers could be used as potential tools to develop virus resistant cotton cultivars (Shweta and Khan, 2014).

In addition to natural miRNAs, Wang et al. (2015a) reported the first comprehensive identification of 30,550 long intergenic non-coding RNA (lincRNA) loci and 4,718 long non-coding natural antisense transcript (lncNAT) loci in *Gossypium* sp., demonstrating their biased expression patterns toward subgenomes and overall higher methylation levels compared to protein coding genes. Several functional long non-coding RNA (lncRNA) candidates, involved in cotton fiber initiation and elongation were identified. For instance, the miR397 generating lncRNAs have an important role in regulating lignin metabolism in domesticated tetraploid cotton fibers (Wang et al., 2015a). All of these natural miRNAs, lncRNA, and lncNAT are important candidate loci to elucidate many important functional questions in cotton that will serve as a base for designing novel RNAi approaches and studies in near future.

## RNAi Cultivars, Field Trials, and Commercialization

Although RNAi has been extensively used and will remain a valuable experimental tool for functional genomics of cotton, RNAi-based cultivar development and its commercialization for cotton farming is in its very early stage. There is limited information on whether the above-mentioned RNAi studies have resulted in a cultivar development, subjected to field trials, and are being targeted for commercialization or not. To our best knowledge, such efforts were covered by only ∼5% of all RNAi studies reviewed herein (**Figure 1B**), as detailed below.

Field trials for two selected *FAD2* cotton RNAi lines were conducted in Narrabri, Australia during 2003/2004 revealing that RNAi lines had high oleic acid content in cottonseed without adverse effects on any of tested key agronomic traits (Liu et al., 2009). There is no information on the commercialization target of these cottonseed oil lines. Rathore and his colleagues (Rathore et al., 2012; Palle et al., 2013) have targeted the commercialization of the ULGCS cotton RNAi lines mentioned above. Small-scale field trials of these ULGCS lines over a period of 3 years demonstrated that the RNAi phenotype was stable under field conditions and plants exhibited sufficient insect pest resistance due to wild-type levels of gossypol and related terpenoids in other plant organs. Most importantly, there were no negative effects of RNAi on either the yield or quality of the fiber with significantly higher (4–8%) oil content in cottonseeds of RNAi lines compared to wild type parental plants. Researchers concluded that “*field trial results confirmed the stability and specificity of the ULGCS trait suggesting that this RNAi-based product has the potential to be commercially viabl*e” (Stokstad, 2006; Rathore et al., 2012; Palle et al., 2013). Field and laboratory trials were conducted in 2015, 2014, and 2013 by Texas A&M University to develop the data package necessary to seek food, feed, and cultivation deregulation of ULGCS biotech events from all relevant U.S. authorities (United States Department of Agriculture [USDA] Portal, 2015).

**FIGURE 2:**
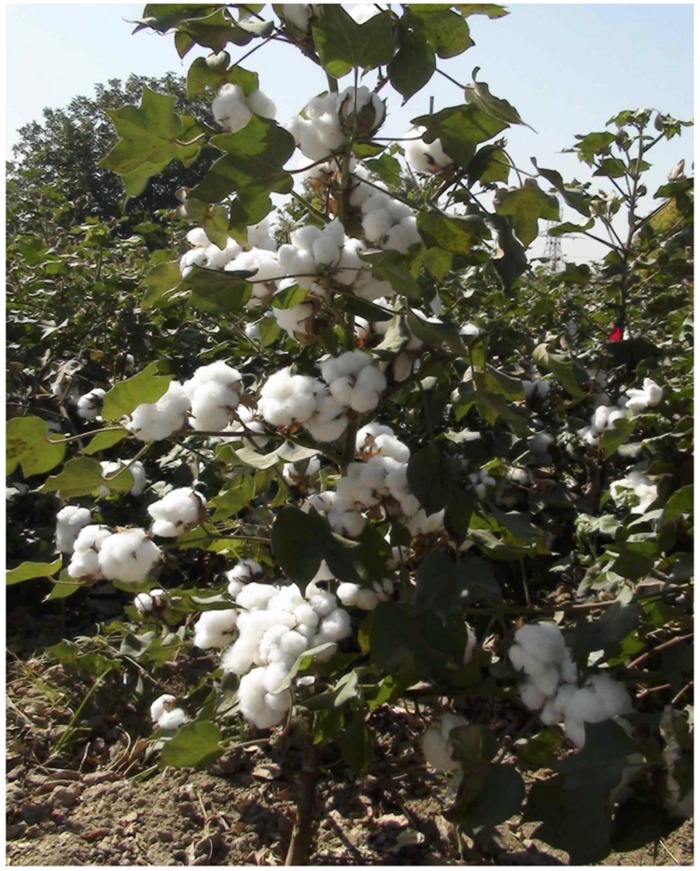
**The plant architecture of *PHYA1* gene-derived and field grown (in 2013, Tashkent farm) novel RNAi cotton cultivar “Porloq-1” (translates as “Shiny-1” or “Great furture-1”, Cotton Outlook, 2014).** This cultivar has been developed through genetic hybridization and multi-generation individual selection from self-pollinated F_2-5_ progeny of the sexual cross between somatically single-cell regenerated RNAi Coker-312 (T-1-7 RNAi family) and Uzbek cotton cultivar AN-Boyovut-2. RNAi of *PHYA1* genes resulted in vigorous shoot and root development, bushy plant architecture, early flowering and increased boll number, early synchronous boll opening and early plant senescence with superior fiber quality parameters among other important morphological and agronomic improvements (refer to Abdurakhmonov et al., 2014 for fiber quality and yield increase details).

**FIGURE 3:**
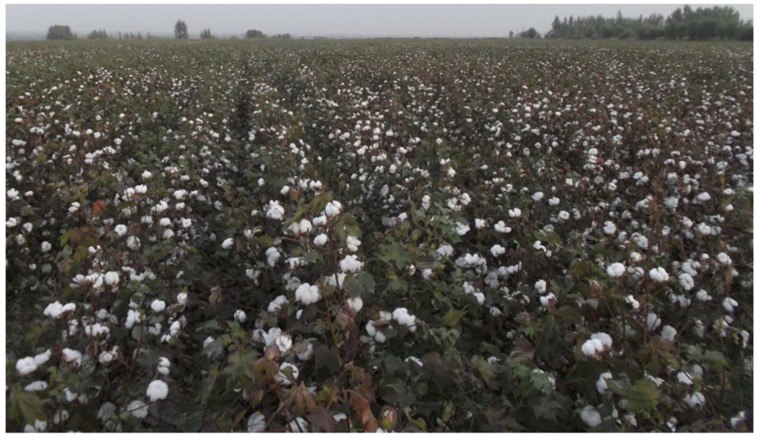
**A general view of large-field trial experimental plot in 2013 for novel Uzbek RNAi cotton cultivar “Porloq-2” derived from the sexual cross between RNAi Coker 312 (T-31 family) and Uzbek commercial Upland cultivar “C-6524” (“Mashhura-N” cotton, Namangan region, Uzbekistan).** This field view demonstrates high-yield potential, early autumn induced rapid-senescence, and simultaneous boll maturity accompanied by increased anthocyanin pigmentation on stems and leaves of RNAi cotton plants under normal solar light condition.

Abdurakhmonov et al. (2014) successfully applied state-of-the-art cotton *PHYA1* RNAi concept and results to the Uzbekistan cotton improvement program. They have demonstrated the transfer of phytochrome-associated RNAi phenotypes from somatically regenerated RNAi Coker-312 to four different (recipient) commercial Upland cotton cultivars via sexual crosses. Efforts effectively converted recipient Upland genotypes to the superior well-adapted RNAi cotton cultivars. As a result, the first generation of novel RNAi cotton cultivar series “Porloq-1,” “Porloq-2,” “Porloq-3,” and “Porloq-4” (translates to “Shiny” or “Great future”) has been developed (**Figures 2** and **3**). These novel RNAi cultivars successfully passed 3 years (2012–2014) of extended field trials across 13 different soil-climatic regions in Uzbekistan (Cotton Outlook, 2014; **Figure 3**). Field trials demonstrated superiority of RNAi cultivars to any traditional Uzbekistan cultivars in terms of fiber quality, adaptiveness to harsh environmental conditions across Uzbekistan, early maturity and significant increase in seed cotton yield and production of increased lint fiber per hectare (Abdurakhmonov et al., 2012b).

There are several positive ramifications associated with the anticipated usage of *PHYA1*-dervied RNAi cultivars by farmers locally and globally that include: (1) opportunity to produce superior, novel RNAi cotton fiber that should have a premium price increase and income per acre, and (2) opportunity to spin a fine-count cotton yarn from any production zone. Furthermore, (3) early flowering and maturity of *PHYA1*-derived RNAi varieties should provide an opportunity for early and quality crop harvest and on-time planting of rotation crops before harsh weather arrives, perfectly suitable and demanding for northernmost cotton growing country like Uzbekistan. RNAi cultivars have (4) increased seed cotton and lint yield per acre – the foremost target and interest of farmers. Most importantly, early observations and ongoing field experiments revealed (5) a better utilization of fertilizers and nutrients (due to increased nitrate reductase activity and strong root system), increased photosynthesis rate, and salt, heat and drought tolerance are added advantages of novel *PHYA1* RNAi varieties. This sufficiently addresses the issue of problematic shortages in irrigation and water deficiencies triggered by forecasted global warming that remains a priority danger for the Central Asian regions (Abdurakhmonov, 2013). The use of cotton’s own gene unlike other existing transgenic technologies of cotton, utilizing foreign genes, this state-of-the-art *PHYA1*-mediated RNAi technology assures (6) ecological safety of RNAi cotton cultivars. A high premium price of cotton fiber and increased yield should allow the expanded cotton production (7) on marginal land and create a new cotton fiber/cloth market; and (8) decrease in cotton planting area provides an opportunity to grow more food crops/plants that help to sustain world agricultural, food security and environment at the regional and global levels.

Production of ‘not yet existed in world fiber market’ novel Upland cotton fiber with 38–41 code (versus possibility of production of fiber with only maximum 35–37 code from existing ordinary Upland cottons) would have a premium price and at least $100 increase in income per acre in Uzbekistan and worldwide. This means that Uzbekistan could earn additional $250 million from its current annual production of one MMT of lint fiber. From this high quality Upland fiber it is possible to spin a fine count 50–70 Ne cotton yarn (against 30-40Ne from ordinary Uplands that Uzbekistan produces). The yarn quality difference itself provides a 10% higher income from these novel RNAi cultivars. Reducing the cotton acreages due to yield and fiber quality improvement of RNAi cultivars would provide opportunity of planting more other food crops ensuring food security for people and sustainability of the environment suffering from cotton production and application of agricultural chemicals.

In 2014, all four RNAi cultivars of “Porloq” series mentioned above were approved for larger field trials in Uzbekistan. RNAi cultivars have been registered through the State List of Approved Agricultural Crops of Uzbekistan. With the purpose of conducting large-scale field trial, RNAi cultivars were planted in over 60,000 hectares (5% of total cotton growing area of Uzbekistan) in the 2015 season in the six major provinces (Namangan, Fergana, Tashkent, Jizzakh, Samarkand, Surkhandarya, and Khorazm) of Uzbekistan. This is to increase RNAi cultivar seeds for upcoming seasons as well as produce larger quantities of “first bales” of RNAi fiber for internal market to test textile quality parameters in larger scale and gather consumer’s opinion (Cotton Outlook, 2014). Further, a collaborative effort between USDA/ARS and Uzbekistan made it possible to transfer the RNAi seeds from Uzbekistan to the USA where USDA partners are field-trialing RNAi lines in the USA environment in 2014/15. Efforts are in progress to mobilize *PHYA1* RNAi effects into several Upland cotton cultivars of the USA and conduct extended field evaluations in 2015/16. These efforts will help in technology transfer among the USA farmers and eventually to other stakeholders of all cotton-growing countries.

## Safety and Risk Assessments

Although RNAi technology is generally perceived as a safer technology for obtaining desired traits, currently RNAi-derived traits are considered as GE products and, therefore, they are also subjected to safety assessment like the products derived from other methods of transgenesis. RNAi activity may not only target genes of interest but could also affect genes with sufficient sequence homology leading to the ‘off-target’ gene silencing with the possible adverse effects on human and animal health or the environment including non-target organisms (NTOs; Casacuberta et al., 2014; Ramon et al., 2014; Roberts et al., 2015). Particularly, the utilization of plant-mediated RNAi as a biological insecticide has raised debates and discussions on its impact to NTOs “*including natural enemies, pollinators, soil decomposers, leaf shredders, wildlife, and fish*” that could possibly be exposed to the plant expressed dsRNAs (Roberts et al., 2015).

Moreover, it should be noted that despite the use of cottons own gene(s) or gene fragment(s), all RNAi hairpin binary vectors currently used for plant transformation, including cotton, have the antibiotic resistance marker (ARM) genes that lately raised great concerns with the use of ARMs in the GMO and its products. However, there is already a long history of safe utilization of ARMs in food and non-food plant products of biotechnology, including cotton. Moreover, there are confirmed evidences of negligibility of the practical impacts of consumption of ARM-containing plant products by humans and animals and horizontal transfer of ARMs from GM crops to environment in natural conditions (Goldstein et al., 2005). Furthermore, cotton is a technical crop and grown mainly for its fiber, making it a more suitable crop for RNAi modifications compared to food crops.

For the assessments of possible risks of RNAi, cotton cultivars and their products there are pre and post-RNAi development tasks. During pre-RNAi cultivar development, an important step is that the designed dsRNA fragment, to be cloned into a hairpin construct, must be thoroughly analyzed *in silico* for the production of all possible siRNA species as well as their putative targets in key consumer or readily exposed organism genomes using the target-searching algorithms against available genome databases. Although there are some limitations due to the lack of genome sequences for non-models and NTOs (Casacuberta et al., 2014), this step could identify the first step risks possibly caused by unintended function of ‘candidate’ dsRNA from a gene of interest. If any such unintended targeting or highly specific off-target matching in consumer genomes is detected, then RNAi development must be stopped, or candidate dsRNA must be changed, fine-tuned/modified and optimized to eliminate the unintended effects. If dsRNA is targeting only the intended function, then the next step is to develop the RNAi cultivar and begin post-RNAi cultivar testing. It includes: (1) the evaluation of the RNAi cultivar for desired trait expression; (2) experimental quantification of the gene-knockdown level of targeted gene expression; and (3) comparative sequencing of siRNA sequence profile before and after RNAi. Although currently not validated within the regulatory framework for food safety assessment, additionally, comparative studying of proteome and metabolome profiles of RNAi cultivar/product may be considered (Ricroch et al., 2011; Clarke et al., 2013; Simó et al., 2014; Wang et al., 2015b). These steps could identify any “off-target” phenotypes, visible side effects, production of unexpected novel siRNA or/and secondary RNA signatures as well as unknown proteome/metabolome components in RNAi cultivar genome and its products. If such novel, unintended, and secondary biomolecules are identified, it should be a warning signal to be cautious with the future use of RNAi product/cultivar. Further, RNAi cultivar products such as cell extracts containing products of RNAi are suggested to be tested (5) in cell/tissue culture and further in animal feeding experiments in model organisms including mammals to assure the safety of RNAi products *in vivo* level. These steps would verify possible biological risks on the utilization of RNAi cultivar and its products or prove its complete safety. Heinemann et al. (2013), has outlined the risk assessment steps, highlighted here.

In Uzbekistan, following the above mentioned risk assessment criteria/guidelines, we started a large, coordinated mega-project for the risk assessment of *PHYA1* RNAi cotton cultivars with involvement of responsible organizations including Cotton Industry Associations, Food Industry Companies, Ministry of Agriculture and Water Resources, Academy of Sciences, Cambridge Center for High Technologies, and Ministry of Health of Uzbekistan. Cotton-derived products including cottonseed oil and feeding products are being comparatively evaluated biochemically and toxicologically via animal feeding experiments in mice.

## Conclusion and Future Perspectives

Thus, replacing the anti-sense technology, RNAi has been proven to be a valuable tool for functional genomics of cotton for the past decade. RNAi revolutionized the discovery of many key functions and biological roles of cotton genes involved in fiber development, fertility and SM, resistance to key biotic and abiotic stresses, oil and seed quality and other important cotton genes as well as in the improvement of key agronomic traits including yield and maturity. Future studies most likely will target the identification and RNAi of more complex, multi-functional, and developmental genes with cross-talking features among many interconnected networks and biochemical pathways of cotton ontogenesis (Abdurakhmonov, 2013) that generate simultaneous improvements of key agronomic traits (Abdurakhmonov et al., 2014). Some efforts highlighted herein demonstrate early examples to show the power of RNAi for cotton improvement that needs to be extended in the future.

The development of RNAi cotton lines targeting core RNAi machinery including proteins with important DNA methylase and demethylase activity [such as DOMAINS REARRANGED METHYLASE 1/2 (DRM1/2), CHROMOMETHYLASE 2/3 (CMT 2/3), ROS1, and DEMETER (DME)] in Upland cotton will aid in elucidating the key regulatory mechanisms in WGD, chromosomal rearrangements, dosage compensation, and evolutionary advantage of being polyploids. Further, screening the epigenetic modulators for specific traits such as fiber and yield and comparing against the genetic standard TM-1 will aid in understanding the epigenetic landscape of Upland cotton.

There is limited information available on downstream stage of RNAi cotton cultivar development, conducted field trials, or targeting for its commercialization. This indicates that possibly RNAi-based “biotech cotton” cultivar development is in its very early stage that requires more attention, effort, investment and partnership of the cotton research community and private seed companies. However, there are some commercially viable and already ongoing, targeted applications of RNAi technologies, which are passing the successful small- or large-scale field trials, and safety/risk assessment studies. These RNAi-based cotton cultivars, highlighted in this paper, present substantial benefit in cotton production with increased seed and oil quality, fiber, and key agronomic trait improvements. These efforts are believed to boost cotton production and its sustainability worldwide in the era of global climate change and increased crop biosecurity threats.

Despite general safety, RNAi based cultivars are subject for risk assessment before commercialization as per available (Heinemann et al., 2013) and highlighted guidelines herein. The existence and use of ARM genes in current RNAi-based cotton cultivars is subject for rising “unjustified” warnings and requests for removal of ARM genes from RNAi cultivars, and this perhaps continues to be a main roadblock for future commercialization of RNAi cotton cultivars. Therefore, there is a need for designing ARM-free RNAi transformation experiments and development of ARM-free RNAi cotton cultivars.

RNA interference studies, reviewed herein, mostly have utilized stable HP or transient VIGS vector constructs. There is a need for application of novel genome modification and editing tools such as artificial microRNA (amiRNA; Liang et al., 2012), short synthetic interfering siRNA oligonucleotides (Higuchi et al., 2009; Abdukarimov et al., 2011), Transcription Activator-like Effector Nucleases (TALENs; Zhang et al., 2013b), and Clustered Regularly Interspaced Short Palindromic Repeats (CRISPRs/Cas9; Larson et al., 2013) system to generate more effective, fine-tuned, native knockdowns/knockouts than currently used RNAi methods. There is no doubt that the cotton research community is already targeting these objectives, having several diploid (Paterson et al., 2012; Wang et al., 2012; Li et al., 2014) and key allotetraploid (Li et al., 2015a; Liu et al., 2015a; Zhang et al., 2015) genome sequences in hand. All of these will require more coordinated efforts, wider international collaborations, larger investment, and understandings of regulatory and stakeholder agencies.

## Author Contributions

IA – coordinated, wrote, revised manuscript; MA, KU, ZB, SES, HR, and US -collected and analyzed world literature, prepared tables and figures, drafted subsections; SS, MU, JY, RP, ED, VS, and SK – critically read and edited manuscript, drafted subsections; GS, SK, AK, HK, KK, SIS, JJ, AA, and AP rigourously edited and approved manuscript.

## Conflict of Interest Statement

Cotton PHYA1 RNAi study mentioned in this review article has been filed for patenting in Uzbekistan (IAP: 20120069), USA (USPTO:13/445696), and internationally (PCT/US13/27801).

The reviewer AD-P and handling Editor declared their shared affiliation, and the handling Editor states that the process nevertheless met the standards of a fair and objective review.
