# Supplementary material for: RNA Interference for Functional Genomics and Improvement of Cotton (Gossypium sp.)
Source: Front Plant Sci. 2016 Feb 22;7:202. doi: 10.3389/fpls.2016.00202 (PMC4762190; doi:10.3389/fpls.2016.00202)
Supplement: Supplementary file 1 [file Table_1.DOCX]

| **Supplementary Table S1. RNAi-mediated gene silencing efforts in *Gossypium* spp.** | | | |  |
| --- | --- | --- | --- | --- |
| **Genes** | **Used methods** | **Caused phenotype** | **References** |  |
| **Fiber development and quality** | | | | |
| Promoter element (*E6*) | Antisense | No discernible phenotypic changes in fiber development | John (1996) |  |
| GTPase Rac (*RAC13*) | Antisense | Decreased levels of H_2_O_2_ affecting the secondary wall formation of fibers | Potikha et al. (1999) |  |
| Stearoyl-acyl-carrier protein Delta 9-desaturase (*GhSAD-1*) and oleoyl-phosphatidylcholine omega 6-desaturase (*GhFAD2-1*) | Antisense | Development of nutritionally improved cotton seed oil | Liu et al. (2000) |  |
| Sucrose synthase (*SUS*) | Antisense | Repressed fiber development without affecting embryo development and seed size | Ruan et al. (2003) |  |
| Actin cytoskleton (*GhACT1*) | Hairpin RNA (hpRNA) | Inhibited fiber elongation | Li et al. (2005) |  |
| Deetiolated2 (*GhDET2*) | Antisense | Inhibited fiber initiation and elongation | Luo et al. (2007) |  |
| Myeloblastosis (*GhMYB109*) | Antisense | Reduced fiber length | Pu et al. (2008) |  |
| Pectate lyase (*GhPEL*) | Antisense | Reduced fiber elongation | Wang et al. (2010a) |  |
| Myeloblastosis (*GhMYB25*) | hpRNA | Shorter fiber and delayed fiber elongation | Machado et al. (2009) |  |
| Arabinoglactan proteins (*GhAGP4*) | hpRNA | Fiber length became significantly shorter and the fiber quality became worse | Li et al. (2010) |  |
| Vacuolar invertase (*GhVIN1*) | hpRNA | Reduced fiber elongation | Wang et al. (2010b); Wang et al. (2014) |  |
| Myeloblastosis (*GhMYB25-like*) | hpRNA | Produced fiberless seeds, but normal trichomes | Walford et al. (2011) |  |
| Homeodomain (*GhHD-1*) | hpRNA | Reduced trichome formation and delayed the timing of fiber initiation | Walford et al. (2012) |  |
| Protodermal factor 1 (*GbPDF1*) | hpRNA | Delayed fiber initiation and produced shorter fibers and lower lint percentage | Deng et al. (2012) |  |
| A microtubule-severing protein *(KATANIN)*  A lipid biosynthesis gene (*WRINKLED1)* | Virus-induced gene silencing (VIGS) | Produced shorter fibers and elevated weight ratio of cottonseed oil  Increased fiber length but reduced oil seed content | Qu et al. (2012) |  |
| A class I TCP transcription factor (*GbTCP*) | hpRNA | Produced shorter fiber, reduced lint percentage, and lowered fiber quality | Hao et al. (2012) |  |
| Sucrose synthase (*GhSusA1*) | hpRNA | Reduced fiber quality and decreased the boll size and seed weight | Jiang et al. (2012) |  |
| Flavanone 3-hydroxylase (*F3H*) | hpRNA | Suppressed fiber development | Tan et al. (2013) |  |
| Repressor of silencing 1 (*ROS1*) | hpRNA | Reduced fiber growth | Jin et al. (2013) |  |
| Fiber-preferential (*GhPIP2*) | hpRNA | Markedly hindered fiber elongation | Li et al. (2013) |  |
| Proline-rich proteins (*GhPRP5*) | hpRNA | Enhanced fiber development | Xu et al. (2013) |  |
| Homeodomain-leucine zipper (HD-ZIP) (*GhHOX3*) | hpRNA | Reduced (>80%) fiber length | Shan et al. (2014) |  |
| Annexin (*GhAnn2*) | hpRNA | Shorter and thinner fibers | Tang et al. (2014) |  |
| Phytochrome A1 (*PHYA1*) | hpRNA | Vigorous root and vegetative growth, early-flowering, significantly improved upper half mean fiber length and an improvement in other major fiber characteristics | Abdurakhmonov et al. (2014) |  |
| Phosphatidylinositol kinase (*PIK*) | VIGS | Decreased fiber length | Liu et al. (2015b) |  |
| α-expansin genes’ truncated version (*GbEXPATR)* | hpRNA | Induced shorter fibers with thicker cell walls | Li et al. (2015b) |  |
| **Fertility and somatic embryogenesis** | | | | |
| High-mobility group box (*GhHmgB*) | hpRNA | Improved tissue proliferation and differentiation | Hu et al. (2011) |  |
| Somatic embryogenesis receptor-like kinase 1 (*GhSERK1*) | hpRNA | Generated a series of male-sterile cotton lines | Shi et al. (2014) |  |
| KORRIGAN (*GhKOR1*) | hpRNA | Resulted in smaller filial tissue, reduced seed weight, various abnormalities in endosperm formation and delayed embryo development | Shang et al. (2015) |  |
| Acyl-CoA N-acyltransferase (*GhACNAT*) | VIGS | Resulted in abnormal anther formation and sterile plants | Fu et al. (2015) |  |
| **Biotic stresses** | | | | |
| Cadinene (*CDN*) syntase | Antisense | Reduced gossypol, hemigossypolone, and heliocides in leaves and seeds | Martin et al. (2003); Benedict et al., 2004 |  |
| (+)-delta-cadinene synthase (*cdn1-C4*) | Antisense | Bacterial blight infection | Townsend et al. (2005) |  |
| Cytochrome P450 gene (*CYP6AE14*) | hpRNA | Retarded the larval growth | Mao et al. (2007) |  |
| Nonrace-specific disease resistance 1 (*GhNDR1*) and MAP kinase kinase 2 (*GhMKK2*) | VIGS | Decreased Verticillium wilt resistance in cotton | Gao et al. (2011a) |  |
| Cytochrome P450 gene (*dsCYP6AE14*) | hpRNA | Acquired enhanced resistance to cotton bollworms | Mao et al. (2011) |  |
| Ve homologous gene (*Gbve1*) | VIGS | Compromised resistance to Verticillium wilt | Zhang et al. (2012) |  |
| Cytochrome P450 gene (*CYP9A14*) | hpRNA | Rendered the larvae more sensitive to the insecticide | Tao et al. (2012) |  |
| Somatic embryogenesis receptor kinases genes (*GhSERK*/*GhBAK1*) | VIGS | Decreased Verticillium wilt resistance in cotton | Gao et al. (2013a) |  |
| Gossypol biosynthesis enzyme gene (*GbCAD1)* | VIGS | Compromised resistance to Verticillium wilt | Gao et al. (2013b) |  |
| ghr-miR482 family | miRNA | Upregulation of several NBS-LRR targets | Zhu et al. (2013) |  |
| Cytochrome P450 gene (*dsCYP6AE14*) | hpRNA | Enhanced resistance to bollworm feeding | Mao et al. (2013) |  |
| Enhanced disease susceptibility 1(*GbEDS1*) | VIGS | Increased susceptibility to *V. dahliae* infection | Su et al. (2014) |  |
| Mitogen-activated protein kinase (*MPK9, MPK13 and MPK25*) | VIGS | Enhanced cotton susceptibility to *V. dahliae* | Zhang et al. (2014) |  |
| Homologous cotton genes involved in basic innate immunity | VIGS | Most of these genes responded to both Verticillium infection and jasmonic acid stimuli | Xu et al. (2014) |  |
| **Abiotic stresses** | | | | |
| Sucrose non-fermenting 1-related protein kinase 2 (*GhSnRK2*) | VIGS | Alleviated drought tolerance | Bello et al. (2014) |  |
| R2R3-type MYB transcription factor (*GbMYB5*) | VIGS | Decreased drought tolerance | Chen et al. (2015) |  |
| Cotton *PHYA1* | hpRNA | Improved drought, salt and heat tolerance | Abdurakhmonov et al. (2014) |  |
| **Seed and oil quality** | | | | |
| Stearoyl-acyl-carrier protein Delta 9-desaturase (*GhSAD-1*) and oleoyl-phosphatidylcholine omega 6-desaturase (*GhFAD2-1*) | Antisense | Resulted in increased levels of stearic and oleic acids | Liu, Singh & Green, (2000) |  |
| Oleoyl-phosphatidylcholine omega 6-desaturase (*FAD2*) | Antisense | Resulted in two-fold increase of the oleic acid level with an accompanying decrease of linoleic acids | Sunilkumar et al. (2005) |  |
| *GhSAD-1* and *GhFAD2-1* | hpRNA | Increased stearic and oleic acids with substantial decrease of palmitic acid content | Liu, Singh & Green, (2002) |  |
| Delta-cadinene synthase | hpRNA | Reduced cottonseed-gossypol levels | Sunilkumar et al. (2006) |  |
| Ultra-low gossypol cottonseed (*ULGCS*) | hpRNA | Reduced cottonseed-gossypol levels | Rathore et al. (2012) |  |
| Ultra-low gossypol cottonseed (*ULGCS*) | hpRNA | Reduced cottonseed-gossypol levels | Palle et al. (2013) |  |
| **Other genes** | | | | |
| Green fluorescent protein (*GFP*) | hpRNA | Efficient silencing of GFP marker gene expression for large-scale screening of gene function and drug target validation | Tang et al. (2004) |  |
| Green fluorescent protein (*m-gfp5-ER* reporter) | hpRNA | siRNA delivery using GFP as a visual marker | Tang et al. (2006) |  |
| Phytochrome (*PHYA1, PHYA2, PHYB*), hypocotyl elongated-5 (*HY5*) | hpRNA | Increased boll quantity, early flowering, vigorous root system | Abdukarimov et al. (2011) |  |
| Cloroplastos alterados 1 (*GrCLA1*) | VIGS | Showed an albino phenotype on true leaves | Gao et al. (2011b) |  |
| Cloroplastos alterados 1 (*GrCLA1*) | VIGS | Changed leaves color to albino | Gao and Shan (2013) |  |
| Cloroplastos alterados 1 (*GrCLA1)*, *GaPDS* and *GaANR* | VIGS | Resulted albino or brownish plant phenotypes | Pang et al. (2013) |  |
| Magnesium chelatase subunit I (*CHLI*) and elongation factor-1α(*EF-1α*) | VIGS | Developed of abnormal leaf phenotypes | Gu et al. (2014) |  |
| Phytoene synthase (*GhPSY*) | VIGS | Highly uniform bleaching of the red color | Cai et al. (2014) |  |
| Anthocyanidin reductase (*GhANR11*) | VIGS | Increase in anthocyanins | Zhu et al. (2015) |  |
| Cotton leaf curl Multan betasatellite (*CLCuMB*) | VIGS | Showed efficient silencing of the target genes | Kumar et al. (2014) |  |
| *AC1* and *AC4* | VIGS | Involved in viral replication and gene silencing suppression | Shweta and Khan (2014) |  |
